# Genetically predicted education attainment in relation to somatic and mental health

**DOI:** 10.1038/s41598-021-83801-0

**Published:** 2021-02-22

**Authors:** Shuai Yuan, Ying Xiong, Madeleine Michaëlsson, Karl Michaëlsson, Susanna C. Larsson

**Affiliations:** 1 Unit of Cardiovascular and Nutritional Epidemiology, Institute of Environmental Medicine, Karolinska Institutet, Nobelsväg 13, 17177 Stockholm, Sweden; 2 Department of Public Health Sciences, Karolinska Institutet, Stockholm, Sweden; 3 Department of Education, Health and Social Studies, Dalarna University, Falun, Sweden; 4 Unit of Medical Epidemiology, Department of Surgical Sciences, Uppsala University, Dag Hammarskjölds Väg 14B, 75185 Uppsala, Sweden

**Keywords:** Environmental social sciences, Diseases, Risk factors

## Abstract

A deeper understanding of the causal links from education level to health outcomes may shed a light for disease prevention. In the present Mendelian randomization study, we found that genetically higher education level was associated with lower risk of major mental disorders and most somatic diseases, independent of intelligence. Higher education level adjusted for intelligence was associated with lower risk of suicide attempts, insomnia, major depressive disorder, heart failure, stroke, coronary artery disease, lung cancer, breast cancer, type 2 diabetes and rheumatoid arthritis but with higher risk of obsessive–compulsive disorder, anorexia nervosa, anxiety, bipolar disorder and prostate cancer. Higher education level was associated with reduced obesity and smoking, which mediated quite an extent of the associations between education level and health outcomes. These findings emphasize the importance of education to reduce the burden of common diseases.

## Introduction

Education level is an important health social determinant and has been proposed as a modifiable risk factor for a number of disorders and diseases, such as depression^1^, age-related cognitive decline^2^, suicide^3^, cardiovascular disease^4^, cancer^5^, and several other diseases^6–8^. However, it is unclear whether the associations are causal and independent of intelligence. Understanding the causal effects of education level on diseases can facilitate the aetiology pathway exploration of diseases as well as development of new strategies for disease prevention. Notwithstanding, randomized controlled trials are ethically and practically infeasible on this topic.

Exploiting genetic variants as instrumental variables for an exposure (i.e., education level), Mendelian randomization (MR) can strengthen the causal inference of an exposure-outcome association^9^. Comparing the risk of disease across individuals who have been classified by their genotype enables the causal effect of an exposure to be estimated with substantially less bias, such as confounding and reverse causality, than in a traditional observational analysis^9^. The rationale for diminished bias in MR studies is that genetic variants are randomly assorted and fixed at conception and therefore largely independent of confounders and cannot be modified by disease development^9^.

Several previous MR studies revealed possible causal associations of genetically higher education level with health outcomes, such as Alzheimer’s disease^10,11^, diabetes^12^, cardiovascular disease^13–15^, cancer^16^, myopia^8^, chronic kidney disease^17^, amyotrophic lateral sclerosis^18^, and longevity^19^. In a Mendelian randomization study based on UK Biobank, however, the pattern of the protective effect of higher genetically predicted education level on a broad range of health endpoints was unclear^20^. Education is an upstream health determinant that influences social and community networks and individual lifestyle factors, thereby affecting the risk of various health outcomes. Higher educational attainment has been established to have direct effects on income, alcohol consumption, and physical activity, and inverse associations with smoking, BMI and sedentary behavior. Although genetically proxied higher education level has been associated with several diseases^20^, whether education exerts causal effects on a wide spectrum of health outcomes remains unknown.

Here, we conducted an MR study to disentangle the causal role of education level from intelligence in major mental and neurological disorders and somatic diseases. A secondary aim was to explore whether intelligence is causally associated with the same health outcomes independently of education. We additionally investigated the associations of education level and intelligence with modifiable health-related risk factors. Given that obesity and smoking influence the risk of many diseases^21–25^, we examined whether these two factors mediate the pathway from education to health outcomes.

## Materials and methods

### Study design

The design and hypothesis of the present study are displayed in Supplementary Fig. 1. We used summary-level data from large genome-wide association studies (GWASs) and genetic consortia (Table 1). Totally, our study included 11 mental and neurological disorders, 19 major somatic diseases, body mass index and cigarette smoking. A systematic review was conducted to find meta-analyses of observational studies of education level and diseases (Supplementary Table 1). All GWASs had been approved by a relevant ethical review board and participants had given informed consent. No individual-level data were used in the present MR study. This MR study was approved by the Swedish Ethical Review Authority.

**Table 1:** Characteristics of included studies of mental disorders, somatic diseases, and health-related risk factors.

Outcome	Cases, No	Controls, No	Population	Study source	Data source
**Mental disorder**					
Anorexia nervosa	16,992	55,525	European	Watson HJ et al.	Psychiatric Genomics Consortium
Anxiety	7016	14,745	European	Otowa T et al.	Psychiatric Genomics Consortium
Bipolar disorder	20,352	31,358	European	Stahl EA et al.	Psychiatric Genomics Consortium
Insomnia	397,959	933,051	European	Jansen PR et al.	CNCR
Major depressive disorder	170,756	329,443	European	Stahl EA et al.	UK Biobank
Obsessive–compulsive disorder	2688	7037	European	IOCDF-GC and OCGAS	Psychiatric Genomics Consortium
Posttraumatic stress disorder	30,000	170,000	Mix	Nievergelt CM et al.	Psychiatric Genomics Consortium
Suicide attempts	6024	44,240	European	Erlangsen A et al.	iPSYCH
Schizophrenia	33,426	54,065	European	Psychiatric Genomics Consortium	Psychiatric Genomics Consortium
**Neurological disease**					
Amyotrophic lateral sclerosis	21,982	41,944	European	Kunkle BW et al.	Project MinE
Alzheimer’s disease	12,577	23,475	European	van Rheenen W et al.	IGAP
**Cardiovascular disease**					
Atrial fibrillation	65,446	522,000	Mix	Roselli C et al.	AFGen
Coronary artery disease	60,801	123,504	Mix	Nikpay M et al.	CARDIoGRAMplusC4D Consortium
Heart failure	7382	387,652	European	Aragam KG et al.	UK Biobank
Total stroke	67,162	454,450	Mix	Malik R et al.	MEGASTROKE Consortium
Any ischemic stroke	60,341	NA	Mix	Malik R et al.	MEGASTROKE Consortium
Large artery stroke	6688	146,392	Mix	Malik R et al.	MEGASTROKE Consortium
Small vessel stroke	11,710	192,662	Mix	Malik R et al.	MEGASTROKE Consortium
Cardioembolic stroke	9006	204,570	Mix	Malik R et al.	MEGASTROKE Consortium
Intracerebral haemorrhage	1545	1481	Mix	Woo D et al.	ISGC
**Cancer**					
Breast cancer	122,977	105,974	Mix	Michailidou K et al.	BCAC
Breast cancer ER+	69,501	NA	Mix	Michailidou K et al.	BCAC
Breast cancer ER−	21,468	NA	Mix	Michailidou K et al.	BCAC
Lung cancer	11,348	15,861	European	Wang Y et al.	ILCCO
Prostate cancer	79,194	61,112	European	Schumacher FR et al.	PRACTICAL Consortium
**Other disease**					
Atopic dermatitis	21,399	95,464	Mix	Paternoster L et al.	EAGLE Consortium
Chronic kidney disease	41,395	439,303	European	Wuttke M et al.	CKDGen Consortium
Fracture	53,184	373,611	European	Morris JA et al.	GEFOS Consortium
Gout	13,179	750,634	Mix	Tin A et al.	GUGC
Inflammatory bowel disease	25,042	34,915	European	de Lange KM et al.	UK IBD consortium
Rheumatoid arthritis	29,880	73,758	Mix	Okada Y et al.	GARNET consortium
Type 2 diabetes	74,124	824,006	European	Mahajan A et al.	DIAGRAM consortium
**Risk factor**					
Body mass index	NA	694,649	Mix	Pulit SL et al.	GIANT consortium
Cigarettes per day	NA	337,334	European	Liu M et al.	GSCAN

*AFGen* Atrial Fibrillation Consortium, *BCAC* Breast Cancer Association Consortium, *CNCR* Center for Neurogenomics and Cognitive Research, *DIAGRAM* The DIAbetes Genetics Replication And Meta-analysis, *EAGLE* The EArly Genetics and Lifecourse Epidemiology, *ER* estrogen receptor, *GARNET* Genetics and Allied research in Rheumatic diseases Networking, *GEFOS* GEnetic Factors for Osteoporosis, *GUGC* The Global Urate Genetics Consortium, *GSCAN* Consortium of Alcohol and Nicotine use, *IGAP* The International Genomics of Alzheimer's Project, *ILCCO* The International Lung Cancer Consortium, *ISGC* International Stroke Genetics Consortium, *NA* Not available, *PRACTICAL* The Prostate Cancer Association Group to Investigate Cancer Associated Alterations in the Genome, *SNP* single-nucleotide polymorphism, *UK IBD consortium* UK Inflammatory Bowel Disease Genetics Consortium.

### Selection of instrumental variables

Instrumental variables for education level and intelligence were identified from GWASs of, respectively, 1,131,881 and 269,867 individuals of European ancestries^26,27^. In total, 1271 and 205 single-nucleotide polymorphisms (SNPs) at the genome-wide significance threshold (*p* < 5 × 10^–8^) were identified to be associated with education level and intelligence, respectively. Independent SNPs (*r*^2^ < 0.01 and clump window > 10 kb) without linkage disequilibrium were proposed as instrumental variables. Linkage disequilibrium among SNPs was calculated based on 1000 genomes LD reference panel (European population) using the PLINK clumping method. Possible palindromic SNPs were excluded. We used 663 and 178 SNPs as instrumental variables for education level and intelligence, respectively. The same instrumental variables were used in the multivariable MR analyses. Education level was defined as number of years of education and was unified across included studies according to an International Standard Classification of Education category. The sample-size-weighted mean of education year was 16.8 years of schooling with a standard deviation (SD) of 4.2 years. For the definition of intelligence, included cohorts extracted a single sum score, mean score, or factor score from a multidimensional set of cognitive performance tests in GWAS with linear model, with the exception of High-IQ/Health and Retirement Study where a logistic regression GWAS was run with “case” status (high intelligence) versus controls (normal intelligence level). All included GWASs adjusted for key covariates, such as age, sex and principal components for ancestry.

### Outcome sources

Summary-level data for the associations of the education- and intelligence-associated SNPs with the outcomes were extracted from large-scale GWASs or genetic consortia. In the present MR study, we included 11 mental and neurological disorders^28–38^, 9 cardiovascular diseases^39–43^, 3 major cancers^44–46^, 7 other diseases^47–53^, body mass index^54^ and cigarette smoking^55^. We did not find proxies for exposure-associated SNPs that were unavailable in the outcome datasets given that the percentage of missing SNPs for most outcomes were minimal and a few missing SNPs was not likely to bias the results based on hundreds of SNPs. Detailed information, such as the number cases and controls, population structure and the source for each outcome, is presented in Table 1. Definitions of the outcomes are presented in Supplementary Table 2.

### Systematic review for meta-analysis of observational studies

A systematic literature search was conducted in the PubMed database before November 1st, 2019 to find meta-analyses of observational studies of education level in relation to diseases studied in the present MR study. We found latest published meta-analysis on 13 diseases and two risk factors, including major depressive disorders^56^, suicide attempts^57^, posttraumatic stress disorder^58^, amyotrophic lateral sclerosis^59^, Alzheimer’s disease^60^, coronary artery disease^61^, heart failure^62^, stroke^63^, breast cancer^64^, prostate cancer^65^, lung cancer^66^, type 2 diabetes^67^, chronic kidney disease^68^ and body mass index^69^. We extracted publication data (PubMed identifier number, the first author's name and year of publication), sample size, and risk estimates with their corresponding confidence intervals. Search strategy and characteristics of included meta-analyses are shown in Supplementary Table 1.

### Statistical analysis

The random-effects inverse-variance weighted method was used to assess the associations of education and intelligence with the outcomes. The weighted median method and MR-Egger regression were used as sensitivity analyses to examine the consistency of results and to detect potential pleiotropy. The weighted median method gives accurate estimates if at least 50% of the instrumental variables are valid^70^. The MR-Egger regression can detect and adjust for pleiotropy albeit rendering low precision of the estimates^71^. The false discovery rate method was used to adjust for multiple testing (Supplementary Tables 3 and 4). For associations that survived multiple testing, we used the multivariable MR method^72^ to disentangle the causal effect of education level on outcomes independent of intelligence and vice versa. For intelligence-adjusted inverse associations that survived multiple testing, we also used the multivariable MR analysis with adjustment for body mass index and smoking to explore the mediation effects of these factors on the associations between education and health outcomes. We performed several multivariable MR analyses to test the mediation effect from education, intelligence, body mass index or smoking, rather than allowing for independent effects as well as mediations by these factors in one MVMR model simultaneously.

Proportions of attenuated effect size were calculated to present the magnitude of mediation effects. Odds ratios (ORs) and 95% confidence intervals (CIs) of diseases and changes of levels of risk factors were scaled to an SD increase in genetically predicted years of education (4.2 years) and intelligence. All statistical analyses were two-sided and performed using the mrrobust package^73^ in Stata/SE 15.0 (StataCorp. 2017. Stata Statistical Software: Release 15. College Station, TX: StataCorp LLC.) and TwoSampleMR^74^ in R Software 3.6.0 (R Core Team. R Foundation for Statistical Computing. Vienna, Austria. 2019. https://www.R-project.org).

### Ethical approval

This MR study was approved by the Swedish Ethical Review Authority.

### Informed consent

All participants included in the genome-wide association studies gave informed consent.

## Results

### Genetically predicted education level and diseases

**Figure 1:**
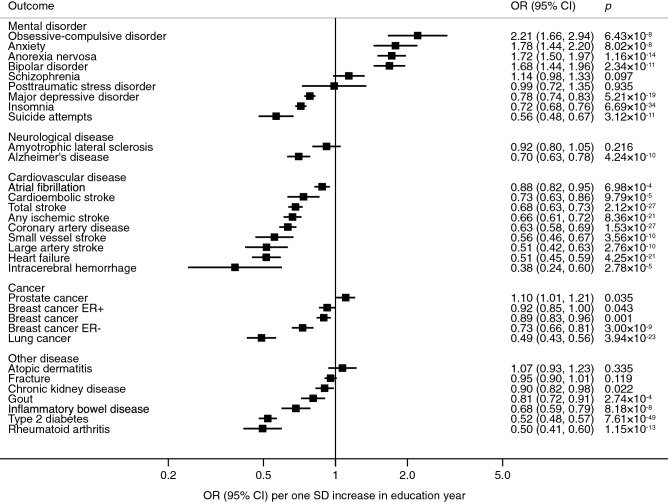
Associations of genetic predisposition to higher education level with health outcomes in univariable MR analyses. *CI* confidence interval, *ER* oestrogen receptor, *IVW* inverse-variance weighted, *OR* odds ratio, *SD* standard deviation.

**Figure 2:**
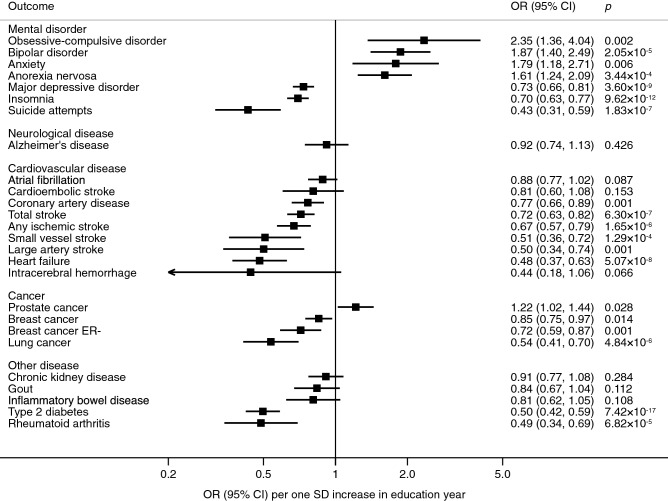
Associations of genetic predisposition to higher education level with health outcomes in multivariable MR analyses with adjustment for genetically predicted intelligence. *CI* confidence interval, *ER* oestrogen receptor, *IVW* inverse-variance weighted, *OR* odds ratio, *SD* standard deviation.

Genetically predicted education level was causally associated with most diseases, including 8 out of 11 mental and neurological disorders, all 9 studied cardiovascular diseases, all 3 studied cancers, and 5 out of 7 other common diseases in the univariable inverse-variance weighted MR analysis (Fig. 1 and Supplementary Table 3). In the multivariable inverse-variance weighted analysis, the associations of education level with Alzheimer’s disease, atrial fibrillation, cardioembolic stroke, intracerebral haemorrhage, chronic kidney disease, gout, and inflammatory bowel disease did not remain after adjustment for intelligence (Fig. 2 and Supplementary Table 3). Results of sensitivity analyses were directionally similar but with wider CIs (Supplementary Table 5).

### Genetically predicted intelligence and diseases

The associations between intelligence and outcomes are presented in Supplementary Tables 4, 6 and 7. Genetically predicted intelligence showed associations with obsessive–compulsive disorder, anorexia nervosa, schizophrenia, insomnia, suicide attempts, Alzheimer’s disease, coronary artery disease, breast cancer (ER−), lung cancer, type 2 diabetes and rheumatoid arthritis in the univariable MR analyses (Supplementary Tables 4 and 6). After adjustment for genetically predicted education level, only the association with schizophrenia persisted (Supplementary Tables 4 and 7).

### Education, intelligence, body mass index and smoking

**Figure 3:**
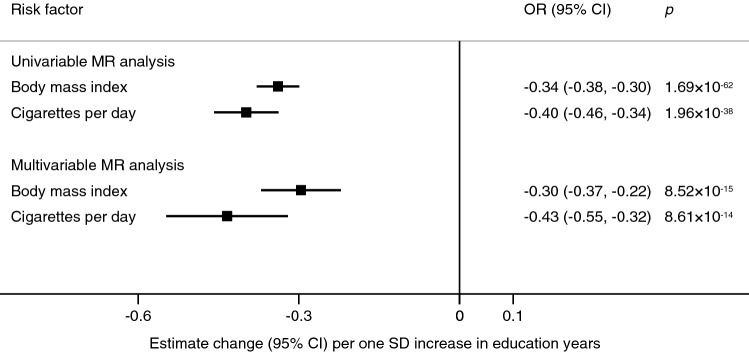
Associations of genetic predisposition to higher education level with body mass index and smoking in MR analyses without and with adjustment for genetically predicted intelligence. *CI* confidence interval, *IVW* inverse-variance weighted, *SD* standard deviation.

Genetically predicted higher education level was associated with lower body mass index and fewer cigarettes smoked per day in the univariable model; the estimates were similar in the intelligence-adjusted model (Fig. 3 and Supplementary Tables 3 and 8). Findings were consistent in sensitivity analyses and no pleiotropy was observed (Supplementary Table 5). Genetically predicted intelligence was not associated with body mass index or smoking (Supplementary Tables 4, 6 and 7).

### Comparison with observational studies

The present MR findings were generally similar in the direction and magnitude to the estimates based on meta-analyses of observational studies (Supplementary Table 9). However, there were discrepancies concerning the effects of education level on suicide attempts, breast cancer and prostate cancer.

### Mediation effects of body mass index and smoking

Table 2 shows the results of mediation analyses after adjusting for body mass index and smoking behaviour in the pathway from education to health outcomes. Although not apparent for all disease outcomes, body mass index and smoking partly mediated most associations between education and diseases. After adjustment for both body mass index and smoking, the direct causal effect of education on the outcomes was substantially attenuated for type 2 diabetes (64%), major depressive disease (44%), heart failure (36%) and coronary artery disease (35%).

**Table 2:** Mediation analysis to disentangle the effects of body mass index and smoking in the pathway from education level to health outcomes.

Outcomes	Total effect of education		Effect after adjusting for BMI			Effect after adjusting for smoking			Effect after adjusting for both		
	OR^a^	95% CI	OR^b^	95% CI	%*	OR^c^	95% CI	%*	OR^d^	95% CI	%*
**Mental disorder**											
Insomnia	0.72	0.68, 0.76	0.75	0.71, 0.80	12	0.74	0.69, 0.79	8	0.77	0.72, 0.82	20
Major depressive disorder	0.78	0.74, 0.83	0.85	0.80, 0.90	35	0.83	0.77, 0.88	25	0.87	0.81, 0.93	44
Suicide attempts	0.56	0.48, 0.67	0.60	0.50, 0.72	12	0.67	0.54, 0.82	31	0.68	0.55, 0.84	33
**Cardiovascular disease**											
Coronary artery disease	0.63	0.58, 0.69	0.67	0.61, 0.73	13	0.72	0.65, 0.79	29	0.74	0.66, 0.82	35
Heart failure	0.51	0.45, 0.59	0.55	0.47, 0.64	11	0.64	0.54, 0.75	34	0.65	0.55, 0.77	36
Total stroke	0.68	0.63, 0.73	0.70	0.64, 0.75	8	0.75	0.69, 0.81	25	0.76	0.69, 0.82	29
Any ischemic stroke	0.66	0.61, 0.72	0.67	0.61, 0.74	4	0.69	0.63, 0.77	11	0.70	0.63, 0.78	14
Large artery stroke	0.51	0.42, 0.63	0.54	0.43, 0.68	8	0.59	0.46, 0.76	22	0.59	0.46, 0.77	22
Small vessel stroke	0.56	0.46, 0.67	0.54	0.44, 0.67	0	0.54	0.43, 0.67	0	0.53	0.42, 0.66	0
**Cancer**											
Breast cancer	0.89	0.83, 0.96	0.92	0.85, 1.00	28	0.93	0.85, 1.01	38	0.94	0.86, 1.03	47
Breast cancer ER−	0.73	0.66, 0.81	0.74	0.66, 0.83	4	0.71	0.63, 0.80	0	0.71	0.63, 0.81	0
Lung cancer	0.49	0.43, 0.56	0.56	0.48, 0.65	19	0.53	0.45, 0.63	11	0.59	0.49, 0.70	26
**Other diseases**											
Rheumatoid arthritis	0.50	0.41, 0.60	0.44	0.36, 0.53	0	0.41	0.34, 0.50	0	0.42	0.34, 0.52	0
Type 2 diabetes	0.52	0.48, 0.57	0.60	0.55, 0.64	22	0.75	0.70, 0.81	56	0.79	0.73, 0.85	64

*BMI* body mass index, *ER* estrogen receptor.

*Percentage of the effect of education on the health outcome that is mediated by body mass index, smoking, or both (Formula: (log(OR_total) − log(OR_adjusted))/log(OR_total) × 100)). We replaced the values with zero for those percentage below zero.

^a^Total effect without any adjustment.

^b^Adjusted for the effect of body mass index.

^c^Adjusted for the effect of smoking (cigarettes per day).

^d^Adjusted for the effects of both body mass index and smoking behaviours.

## Discussion

In the present MR study, genetic predisposition to higher education level was causally associated with the majority of major health outcomes, body mass index and smoking. Specifically, genetic predisposition to higher education level, independent of intelligence, was associated with lower risk of major depressive disorder, insomnia, suicide attempts, coronary artery disease, stroke, heart failure, breast cancer, lung cancer, type 2 diabetes, and rheumatoid arthritis. Conversely, higher education level was associated with higher risk of obsessive–compulsive disorder, bipolar disorder, anxiety, anorexia nervosa and prostate cancer. Genetically predicted higher intelligence, independent of education, was inversely related to schizophrenia. Body mass index and smoking displayed strongest mediation effects observed for type 2 diabetes, major depressive disease, heart failure and coronary artery disease.

### Comparison with previous studies

Our findings are broadly in line with a vast body of observational studies showing a protective association of high educational level on major depressive disorder^56^, Alzheimer’s disease^60^, coronary heart disease^61^, heart failure^62^, stroke^63^, lung cancer^66^, type 2 diabetes^67^, chronic kidney disease^68^, and obesity^69^. However, for suicide attempts, posttraumatic stress disorder, breast cancer and prostate cancer, our MR findings differ from observational findings. The discrepancies might be attributed by reverse causality in the observational studies, heterogeneity and small sample sizes in the meta-analyses. A substantial heterogeneity (*I*^2^ = 85%; *p* < 0.001) was observed among included observational studies in the meta-analysis of breast cancer^64^, and the sample size was small for prostate cancer^65^. Some studies have proposed that the higher risk of prostate cancer among men with high education level was driven by higher prostate-specific antigen screening rate among educated men compared with men with low education level^75^. With regard to the inverse association of higher education level with breast cancer, the association may in part be mediated by reproductive or hormone-related factors, or other health behaviours such as healthier diet and physical activity. We are not aware of any previous MR studies on education or intelligence in relation to prostate or breast cancer, but a protective causal effect of higher education on lung cancer risk has been reported recently^16^.

Previous MR studies showed a protective effect of higher educational level on Alzheimer’s disease^10,11^, type 2 diabetes^12^, cardiovascular disease^13–15^, lung cancer^16^, myopia^8^, chronic kidney disease^17^ and amyotrophic lateral sclerosis^18^. The present study using a larger body of SNPs as instrumental variables more precisely verified these findings and expanded the map of other health benefits of improved education level. Notably, the effects of high education level in some previous studies might be influenced by high intelligence given the tight phenotype and genetic correlation between intelligence and education level. In the present study, we used multivariable MR analysis to assess the direct effect of education level that is not mediated via intelligence. For Alzheimer’s disease, we found that higher intelligence rather than education level may be the protective factor. In a previous MR study of the direct effect of education and intelligence on certain health outcomes, including diabetes, hypertension, heart attack, total stroke, total cancer, and depression, no significant association with education or intelligence was observed despite significant or suggestive associations of genetically predicted education with potential risk factors^20^. Findings of other MR studies of education level in relation to obesity^76^ and cigarette smoking^77^ are consistent with our findings.

### Possible mechanisms

Based on results of the present MR study and previous observational studies, there are three major possible pathways linking education level to health outcomes: (1) modifiable risk factors largely mediates the educational effects on diseases^15,78^; (2) there may be direct effects from education-related brain structures or function change via gene methylation, gene silencing etc.^79–81^, especially for mental and neurological disorders; and (3) subjective well-being, happiness and meaning of life influenced by education level exerts effects on mental and somatic diseases directly or indirectly^82–85^. Education, as measured in this study, can be defined as an institutionalized form of social resource, and more specifically a form of cultural capital drawing on the terminology of the French sociologist Pierre Bourdieu. Related forms of cultural capital emerge as objectivized resources—such as books, art or scientific tools—or incorporated resources, such as knowledge, attitudes and practices^86,87^. Our study shows that education is a health relevant cultural capital whilst intelligence is not to the same degree related with health and risk of disease.

Observational studies have found that the associations between education level and diseases attenuated largely after adjustment for health-related risk factors. Compared with unadjusted model, the risk of cardiovascular diseases of low education attainment attenuated around 30–45% in statistical models adjusted for multiple risk factors^88,89^. In the present study, genetically predicted education level was associated with a favourable risk factor profile: with improved smoking behaviours as well as lower adiposity, which might mediate associations between education level and diseases. By conducting mediation analysis, we showed that body mass index and smoking behaviour partly or entirely mediated the pathway from education level to several health outcomes.

Previous studies have found that low education level might influence the changes in biochemical response and risk-related brain function, such as inflammation^79^, cardiometabolic traits^80^, and amygdala reactivity^81^, via gene methylation, thereby influencing disease risk. In addition, genetic studies have also revealed that improvement of subjective well-being^82,83^, happiness^82,83^, meaning of life^84^, social interaction^85^, possibly derived from high education level benefited human health directly and indirectly (e.g. influencing brain morphology, central nervous system and adrenal/pancreas tissues). There are other possible explanations, like followings: education level also could modify the risk of health outcomes through other diseases (comorbidity), the use of health care services, neighbourhood environment, occupations, income and marital status, which were amenable if education level was increased.

The results indicate that more than knowledge itself is affecting how people live their life, for instance through pathways regarding reduced smoking habits among highly educated people. Therefore, we should consider further explanations, such as the relationship between high education on the one hand and the status and resources that follow it, on the other, which could by itself have a positive health effect on the individual. A further explanation assumes that it is the process itself that can be associated with increased well-being. That is, the process of taking part of and acquiring external knowledge rather than remaining with one's own innate thinking or being kept oblivious. Should only a fraction of the disease burden be explained by this process of mental activity—given that education leads to a different kind of thinking, which is supported by the present study in that health is affected regardless of intelligence level—then increased knowledge through education may lead to longevity through mechanisms beyond health literacy pathways of late-onset diseases and beyond the influence of social and material factors.

### Strengths

The present study is the first study that comprehensively investigated the causal effects of education and intelligence on a very broad range of major disease outcomes using genetic data from large-scale GWASs and genetic consortia. We used SNPs deriving from a larger GWAS with around 1.1 million individuals as instrumental variables for education level, thereby assuring adequate statistical power to detect weak associations. In addition, we disentangled the independent effect of education level from intelligence using a multivariable MR approach. Thus, it is a straightforward approach to estimate the possible health benefits from education promotion among general population. We used mediation analysis to reveal the roles of body mass index and smoking behaviour as mediators in the pathway from education level to health outcomes. Even though there were genetic data for certain outcomes from GWASs with trans-ancestry populations, the majority of included participants were individuals with European ancestry thereby diminishing population stratification bias. However, population confinement limited the transferability of the present findings to populations of non-European ancestries.

### Limitations

The major limitation of the present study is the possible unbalanced horizontal pleiotropy aroused from used genetic variants marking more generic biological pathways. It has been found that the lead SNPs related to education level and intelligence are significantly overexpressed in the central nervous system, such as hippocampus and cerebral cortex, but not in other organs^26^. For cardiovascular disease, cancers and other physical diseases, we can minimize the possibility of pleiotropy from the global or systemic measures of fitness (such as mitochondrial function). It is more likely to conclude that the potential pleiotropy might exert a large to moderate effect via predominantly neurological pathways (for example, behaviours associated with obesity or smoking) for somatic diseases. In this scenario, the vertical pleiotropy would not bias the total causal effect by a higher educational level on disease development. With regard to mental and neurological disorders, although gene overwhelmingly expressed in the brain or central nervous system, studies found no, or at most a small, genetic correlation between lower education attainment and mental and neurological disorders by using bivariate genomic-relationship-matrix restricted maximum likelihood analysis^1^. Thus, the associations between education level and mental or neurological diseases were not mainly because of measurable pleiotropic genetic effects, but because of education-related environmental factors. In addition, from a statistical perspective, we detected almost no pleiotropy in the results of MR-Egger regression and the estimates were consistent through sensitivity analyses, which indicated a negligible distortion by pleiotropy. Intergenerational effects from parents for certain disease, such as coronary artery diseases and type 2 diabetes, could not be assessed by using the data in the present MR study.

There was sample overlap in some analyses, which might have resulted in model overfitting if the SNP-education associations were estimated in studies that were included in the outcome dataset. In addition, the present MR study based on summary-level data could not assess potential non-linear associations of genetically predicted education level and intelligence with outcomes. The effect size of our MR findings could not be quantitatively comparable to other studies given that we scaled the associations to one standard deviation increase in genetically predicted education and intelligence, which might differ from study to study.

In summary, the present MR study strengthened the evidence of protective role of high education level on the majority of mental disorders and somatic diseases independent of intelligence. Body mass index and smoking partly mediated several of the associations between education level and health outcomes.

## Supplementary Information

Supplementary Information.

## Data Availability

The datasets analysed in this study are publicly available summary statistics.
