# Supplementary material for: Genetically predicted education attainment in relation to somatic and mental health
Source: Sci Rep. 2021 Feb 22;11:4296. doi: 10.1038/s41598-021-83801-0 (PMC7900220; doi:10.1038/s41598-021-83801-0)
Supplement: Supplementary file 1 — Supplementary Information. [file 41598_2021_83801_MOESM1_ESM.docx]

**Supporting material**

**Genetically predicted education attainment in relation to somatic and mental health**

Shuai Yuan, Ying Xiong, Madeleine Michaëlsson, Karl Michaëlsson, Susanna C. Larsson

**Supplementary Table 1.** Characteristics of included meta-analyses of observational studies and search strategy

**Supplementary Table 2**. Definitions of outcomes in genome-wide association studies

**Supplementary Table 3.** Results of false discovery rate analysis for associations with genetically predicted education level

**Supplementary Table 4.** Results of false discovery rate analysis for associations with genetically predicted intelligence

**Supplementary Table 5.** Associations of genetically predicted education years with diseases based on the weighted median and MR-Egger

**Supplementary Table 6.** Associations of genetically predicted intelligence with diseases, body mass index and smoking

**Supplementary Table 7.** Associations of genetically predicted intelligence with outcomes after adjustment for genetically predicted education level

**Supplementary Table 8.** Associations of education level with body mass index and smoking in the weighted median and MR-Egger models

**Supplementary Table 9.** Comparison of the results of the present MR study with those of meta-analysis of observational studies

**Supplementary Figure 1.** Study design and hypothesis

**Supplementary Table 1.** Characteristics of included meta-analyses of observational studies and search strategy

| **Outcome** | **PMID** | **First author** | **Year** | **Sample size** | **Cases** | **Controls** | **Effect size^a^** | **SE** |
| --- | --- | --- | --- | --- | --- | --- | --- | --- |
| Major depressive disorder | 20565049 | Chang-Quan H | 2010 | 50 988 | 9494 | 41 494 | 0.67 | 0.08 |
| Alzheimer's disease | 26294005 | Xu W | 2015 | >5000 | NA | NA | 0.77 | 0.04 |
| Suicide attempts | 22800121 | Li Y | 2012 | 2954 | NA | NA | 1.24 | 0.22 |
| Amyotrophic lateral sclerosis | 27377857 | Wang MD | 2017 | NA | NA | NA | 0.49 | 0.06 |
| Posttraumatic stress disorder | 29292778 | Tang B | 2017 | 68 685 | 11 963 | 56 722 | 0.81 | 0.03 |
| Coronary artery disease | 28406328 | Khaing W | 2017 | NA | NA | NA | 0.74 | 0.07 |
| Heart failure | 30295783 | Potter EL | 2019 | 6 308 006 | 104 217 | 6 203 789 | 0.60 | 0.07 |
| Total stroke | 28410350 | McHutchison CA | 2017 | 2 737 522 | 164 683 | 2 572 839 | 0.74 | 0.03 |
| Breast cancer | 31479033 | Dong JY | 2019 | >10 million | 194 654 | NA | 1.22 | 0.04 |
| Prostate cancer | 30029628 | Brown CR | 2018 | >14 736 | NA | NA | 0.63 | 0.09 |
| Lung cancer | 19184626 | Sidorchuk A | 2009 | 2 562 221 | 27 395 | 2 534 826 | 0.61 | 0.05 |
| Type 2 diabetes | 29558518 | Bellou V | 2018 | 255 445 | 20 649 | 2 34 796 | 0.71 | 0.03 |
| Chronic kidney disease | 29437863 | Zeng X | 2018 | 193 226 | NA | NA | 0.83 | 0.04 |
| Body mass index | 30654313 | He J | 2019 | 164 049 | NA | NA | -0.11 | 0.02 |

^a^ The effect size is presented by odds ratio for binary outcomes and beta for continuous outcomes.

**Search strategies:**

# **Study design**: "Meta-Analysis" [Publication Type] or meta-analysis[tiab] or meta analysis[tiab] or "Systematic Review" [Publication Type] or systematic review[tiab]

**Exposure**: "Education"[Mesh] or education[tiab] or "Social Class"[Mesh] or socioeconomic[tiab]

**Outcome**

**Major depressive disorder**: "Depressive Disorder, Major"[Mesh] or Major Depressive Disorder[tiab] or Involutional Psychoses[tiab] or Involutional Psychosis[tiab] or Involutional Paraphrenia[tiab] or Involutional Depression[tiab] or Involutional Melancholia[tiab] or "Depression"[Mesh] or depression[tiab]

**Insomnia**: "Sleep Initiation and Maintenance Disorders"[Mesh] or insomnia[tiab] or Sleeplessness[tiab]

**Suicide attempts**: "Suicide, Attempted"[Mesh] or attempted suicide[tiab] or suicide attempt[tiab]

**Bipolar disorder**: "Bipolar Disorder"[Mesh] or Bipolar Disorders[tiab] or Manic Depressive Psychosis[tiab] or Bipolar Affective Psychosis[tiab] or Mania[tiab] or Manic State[tiab] or Bipolar Depression[tiab] or Manic Disorder[tiab]

**Schizophrenia**: "Schizophrenia"[Mesh] or Schizophrenia[tiab]

**Anxiety**: "Anxiety"[Mesh] or anxiety[tiab]

**Anorexia nervosa**: "Anorexia Nervosa"[Mesh] or Anorexia Nervosa[tiab]

**Posttraumatic stress disorder**: "Stress Disorders, Post-Traumatic"[Mesh] or PTSD[tiab] or Posttraumatic Neuroses[tiab] or Posttraumatic Stress Disorder[tiab]

**Obsessive-compulsive disorder**: "Obsessive-Compulsive Disorder"[Mesh] or Obsessive Compulsive Disorder[tiab] or Obsessive-Compulsive Neuroses[tiab]

**Alzheimer's disease**: "Alzheimer Disease"[Mesh] or Alzheimer disease[tiab] or Senile Dementia[tiab] or Alzheimer Type Dementia[tiab] or Alzheimer Sclerosis[tiab] or Alzheimer Syndrome[tiab] or Alzheimer Dementia[tiab] or Familial Alzheimer Disease (FAD)[tiab]

**Amyotrophic lateral sclerosis**: "Amyotrophic Lateral Sclerosis"[Mesh] or Amyotrophic Lateral Sclerosis[tiab]

**Coronary artery disease**: "Coronary Artery Disease"[Mesh] or Coronary Artery Diseases[tiab] or Coronary Arteriosclerosis[tiab] or Coronary Arterioscleroses[tiab] or Coronary Atheroscleroses[tiab] or Coronary Atherosclerosis[tiab]

**Atrial fibrillation**: "Atrial Fibrillation"[Mesh] or Atrial Fibrillation[tiab] or Auricular Fibrillation[tiab]

**Heart failure**: "Heart Failure"[Mesh] or Cardiac Failure[tiab] or Heart Decompensation[tiab] or Myocardial Failure[tiab]

**Stroke**: "Stroke"[Mesh] or stroke[tiab]

**Intracerebral hemorrhage**: "Cerebral Hemorrhage"[Mesh] or Cerebrum Hemorrhage[tiab] or Intracerebral Hemorrhage[tiab]

**Breast cancer**: "Breast Neoplasms"[Mesh] or Breast Neoplasm[tiab] or Breast Tumor[tiab] or Breast Cancer[tiab] or Cancer of Breast[tiab] or Breast Carcinoma[tiab]

**Prostate cancer**: "Prostatic Neoplasms"[Mesh] or Prostate Neoplasm[tiab] or Prostatic Neoplasm[tiab] or Prostate Cancer[tiab] or Prostatic Cancer[tiab]

**Lung cancer**: "Lung Neoplasms"[Mesh] or Lung Neoplasm[tiab] or Pulmonary Neoplasm[tiab] or Lung Cancer[tiab] or Pulmonary Cancer[tiab]

**Type 2 diabetes**: "Diabetes Mellitus, Type 2"[Mesh] or NIDDM[tiab] or Noninsulin Dependent Diabetes Mellitus[tiab] or Type 2 Diabetes[tiab]

**Chronic kidney disease**: "Renal Insufficiency, Chronic"[Mesh] or Chronic Renal Insufficiency[tiab] or Chronic Kidney Insufficiency[tiab] or Chronic Kidney Disease[tiab] or Chronic Renal Disease[tiab]

**Fracture**: "Fractures, Bone"[Mesh] or fracture[tiab] or Broken Bone[tiab]

**Gout**: "Gout"[Mesh] or gout[tiab]

**Rheumatoid arthritis**: "Arthritis, Rheumatoid"[Mesh] or Rheumatoid Arthritis[tiab]

**Inflammatory bowel disease**: "Inflammatory Bowel Diseases"[Mesh] or Inflammatory Bowel Disease[tiab]

**Atopic dermatitis**: "Dermatitis, Atopic"[Mesh] or Atopic Dermatitis[tiab] or Atopic Neurodermatitis[tiab] or Disseminated Neurodermatitis[tiab] or Atopic Eczema[tiab] or Infantile Eczema[tiab]

**Body mass index**:"Body Mass Index"[Mesh] or body mass index[tiab]

**Smoking**: "Smoking"[Mesh] or smoking[tiab]

**Supplementary Table 2**. Definitions of outcomes in genome-wide association studies

| **Outcome** | **PMID** | **Year** | **Definition** |
| --- | --- | --- | --- |
| Bipolar disorder | 31043756 | 2019 | Cases were met international consensus criteria (DSM-IV, ICD-9 or ICD-10).  Control in most samples were for the absence of lifetime psychiatric disorders. |
| Schizophrenia | 29906448 | 2018 | Diagnosis was in accordance with the Diagnostic and Statistical Manual of Mental Disorders-Version IV(DSM-IV) or International Classification of Diseases, 10th Revision (ICD-10) criteria.  Controls were recruited from the Betula study, an ongoing longitudinal, prospective,  population-based study from the same geographic area (North Sweden) that is studying  aging, health, and cognition in adults. |
| Major depressive disorder | 30718901 | 2019 | Broad depression (self-reported past help-seeking for problems with “nerves, anxiety, tension or depression), probable major depressive disorder (MDD) (self-reported depressive symptoms with associated impairment), and International Classification of Diseases (ICD, version 9 or 10)-coded MDD. |
| Alzheimer’s disease | 30820047 | 2019 | clinical confirmation (NINCDS-ADRDA/DSMIV-V criteria or Clinical Dementia Rating (CDR) ≥ 1), autopsy-confirmation |
| Obsessive-compulsive disorder | 28761083 | 2018 | DSM-IV criteria for OCD |
| Amyotrophic lateral sclerosis | 27455348 | 2016 | 1994 El-Escorial Criteria, EFNS Consensus criteria, revised El-Escorial Criteria (2000) |
| Posttraumatic stress disorder | 31594949 | 2019 | DSM-IV PTSD, UCLA PTSD Reaction Index, The PTSD Symptom Scale(PSS-I), DSM-V, CAPS Life Event Checklist, PRIM-PTSD, ICD-10 (F43.1), PTSD Symptom Scale (PSS), Impact of Events Scale-Revised (IES-R), SCID, DSM-IIl-R PTSD Criteria, Trauma Screening Questionnaire (TSQ), PTSD checklist(PCL), self-report,  The majority of controls were trauma exposed. |
| Anxiety | 26754954 | 2016 | DSM-IV criteria |
| Insomnia | 30804565 | 2019 | Self-report sleep questionnaire data  “Do you have trouble falling asleep at night or do you wake up in the middle of the night?”  Insomnia cases were defined as participants who answered this question with “usually”, while participants answering “never/rarely” or “sometimes” were defined as controls. |
| Anorexia nervosa | 31308545 | 2019 | ICD-10 diagnosis of F50.0 or F50.1, DSM-IV-TR. |
| Suicide Attempts | 30116032 | 2018 | Individuals in the iPSYCH sample who at some point prior to December 31, 2012 had been recorded with one or more incidents of non-fatal suicide attempts were considered as cases. |
| Coronary artery disease | 26343387 | 2015 | A CAD qualifying event captured by the electronic databases at any time after Jan 1, 1999 including MI, angina with at least one angiographic stenosis of >50%, or revascularization procedure in men 18 to 45 or women 18 to 55 years of age at the time of the event; MONICA criteria in the Icelandic Myocardial infarction registry; validated history of MI and were verified by hospital records; under the age of 65 with a confirmed primary MI within the preceding 3-36 months; had at least one of myocardial infarction, coronary artery bypass graft, percutanaeous intervention or a stenosis of at least 50% in at least one epicardial vessel; ICD-10; identified following their admission for acute treatment of MI or in cardiac rehabilitation clinics; with first-ever CAD event defined as fatal and non-fatal myocardial infarction, unstable angina or coronary revascularization; had a history of MI, unstable or stable angina, coronary artery bypass grafting, or angioplasty; |
| Stroke | 29531354 | 2018 | Stroke was defined according to the World Health Organization (WHO), i.e. rapidly developing signs of focal (or global) disturbance of cerebral function, lasting more than 24 hours or leading to death with no apparent cause other than that of vascular origin. Strokes were defined as ischemic stroke (IS) or intracerebral hemorrhage (ICH) based on clinical and imaging criteria. IS was further subdivided into the following categories mostly using the Trial of Org 10172 in Acute Stroke Treatment (TOAST) criteria): i) large vessel ischemic stroke (LV-IS); ii) cardioembolic ischemic stroke (CE-IS); iii) small vessel ischemic stroke (SV-IS). |
| Heart failure | 30586722 | 2018 | The presence of self-reported HF/pulmonary edema or cardiomyopathy at any visit; or an International Classification of Diseases, 10th Revision (ICD-10) or International Classification of Diseases, 9th Revision (ICD-9) billing code indicative of heart/ventricular failure or a cardiomyopathy of any cause. Individuals with a diagnosis of hypertrophic cardiomyopathy, as ascertained by self-report or by pertinent ICD-10 codes, were excluded |
| Atrial fibrillation | 29892015 | 2018 | Individuals who had ever had AF based on any ECG available, ICD-9 code for AF, first diagnosis ≤12 months or less before randomization) and at least two stroke risk factors as codified in the CHA2DS2-VASc score, have coronary artery disease with at least 75% stenosis of at least one coronary segment and served as cases, diffusion weighted imaging (DWI) completed within 48 hours after symptom onset |
| Intracerebral hemorrhage | 24656865 | 2014 | A new and acute neurological deficit with compatible brain imaging (computed tomography or magnetic resonance imaging) showing the presence of intraparenchymal bleeding.  Control subjects were ICH-free individuals enrolled from the same population that gave rise to the case subjects at each participating study site, aged >55 years (GOCHA) and >18 years (GERFHS and ESs). |
| Breast cancer | 29059683 | 2017 | Breast cancer diagnosis |
| Prostate cancer | 29892016 | 2018 | ICD-10 |
| Lung cancer | 24880342 | 2014 | International Classification of Diseases for Oncology (ICD-O) or World Health Organisation (WHO) coding |
| Type 2 diabetes | 30297969 | 2018 | Diagnostic fasting glucose (≥7), fasting glucose (≥11.1), HbA1c levels (HbA1c ≥6.5%), hospital discharge diagnosis, use of oral diabetes medication or self-report, ICD-9 codes |
| Gout | 31578528 | 2019 | Self-report, use of urate-lowering medications or International Statistical Classification of Diseases and Related Health Problems (ICD) codes |
| Rheumatoid arthritis | 24390342 | 2014 | The 1987 criteria of the American College of Rheumatology for RA diagnosis, diagnosed with RA by a professional rheumatologist |
| Inflammatory bowel disease | 28067908 | 2017 | accepted endoscopic, histopathological and radiological criteria |
| Fracture | 30598549 | 2019 | ICD-10 codes, questionnaire-based self-reported |
| Chronic kidney disease | 31152163 | 2019 | clinical diagnosis (phenotype was defined as an eGFR below 60 ml min^–1^ per 1.73 m^2^) |
| Atopic dermatitis | 26482879 | 2015 | Web-based questionnaire for medical history: |
| Body mass index | 30239722 | 2019 | NA |
| Cigarettes per day | 30643251 | 2019 | The average number of cigarettes smoked per day  For studies that collected a quantitative measure of cigarettes per day, where the respondent is free  to provide any integer (e.g., 13 cigarettes per day) responses were binned as follows.  a. 1 = 1-5  b. 2 = 6-15  c. 3 = 16-25  d. 4 = 26-35  e. 5 = 36+ |

**Supplementary Table 3.** Results of false discovery rate analysis for associations with genetically predicted education level

|  |  | **Univariable MR** | | **MVMR with intelligence adjusted** | |
| --- | --- | --- | --- | --- | --- |
| **Class** | **Outcome** | ***p-value*** | **Result** | ***p-value*** | **Result** |
| Mental disorder | Obsessive-compulsive disorder | 6.43E-08 | TRUE | 2.00E-03 | TRUE |
| Mental disorder | Anorexia nervosa | 1.16E-14 | TRUE | 3.44E-04 | TRUE |
| Mental disorder | Bipolar disorder | 2.34E-11 | TRUE | 2.05E-05 | TRUE |
| Mental disorder | Schizophrenia | 9.70E-02 | FALSE | - | - |
| Mental disorder | Anxiety | 8.02E-08 | TRUE | 6.00E-03 | TRUE |
| Mental disorder | Posttraumatic stress disorder | 9.35E-01 | FALSE | - | - |
| Mental disorder | Major depressive disorder | 5.21E-19 | TRUE | 3.60E-09 | TRUE |
| Mental disorder | Insomnia | 6.69E-34 | TRUE | 9.62E-12 | TRUE |
| Mental disorder | Suicide attempts | 3.12E-11 | TRUE | 1.83E-07 | TRUE |
| Neurological disease | Alzheimer's disease | 4.24E-10 | TRUE | 4.26E-01 | FALSE |
| Neurological disease | Amyotrophic lateral sclerosis | 2.16E-01 | FALSE | - | - |
| Cardiovascular disease | Coronary artery disease | 1.53E-27 | TRUE | 1.00E-03 | TRUE |
| Cardiovascular disease | Atrial fibrillation | 6.98E-04 | TRUE | 8.70E-02 | FALSE |
| Cardiovascular disease | Heart failure | 4.25E-21 | TRUE | 5.07E-08 | TRUE |
| Cardiovascular disease | Total stroke | 2.12E-27 | TRUE | 6.30E-07 | TRUE |
| Cardiovascular disease | Any ischemic stroke | 8.36E-21 | TRUE | 1.65E-06 | TRUE |
| Cardiovascular disease | Large artery stroke | 2.76E-10 | TRUE | 1.00E-03 | TRUE |
| Cardiovascular disease | Small vessel stroke | 3.56E-10 | TRUE | 1.29E-04 | TRUE |
| Cardiovascular disease | Cardioembolic stroke | 9.79E-05 | TRUE | 1.53E-01 | FALSE |
| Cardiovascular disease | Intracerebral hemorrhage | 2.78E-05 | TRUE | 6.60E-02 | FALSE |
| Cancer | Breast cancer | 1.14E-03 | TRUE | 1.40E-02 | TRUE |
| Cancer | Breast cancer ER+ | 4.27E-02 | FALSE | - | - |
| Cancer | Breast cancer ER- | 3.00E-09 | TRUE | 1.00E-03 | TRUE |
| Cancer | Prostate cancer | 3.50E-02 | TRUE | 2.80E-02 | TRUE |
| Cancer | Lung cancer | 3.94E-23 | TRUE | 4.84E-06 | TRUE |
| Other disease | Type 2 diabetes | 7.61E-49 | TRUE | 7.42E-17 | TRUE |
| Other disease | Chronic kidney disease | 2.15E-02 | TRUE | 2.84E-01 | FALSE |
| Other disease | Fracture | 1.19E-01 | FALSE | - |  |
| Other disease | Gout | 2.74E-04 | TRUE | 1.12E-01 | FALSE |
| Other disease | Rheumatoid arthritis | 1.15E-13 | TRUE | 6.82E-05 | TRUE |
| Other disease | Inflammatory bowel disease | 2.30E-05 | TRUE | 1.08E-01 | FALSE |
| Other disease | Atopic dermatitis | 3.35E-01 | FALSE | - | - |
| Risk factor | Body mass index | 1.69E-62 | TRUE | 8.52E-15 | TRUE |
| Risk factor | Cigarettes per day | 1.96E-38 | TRUE | 8.61E-14 | TRUE |

**Supplementary Table 4.** Results of false discovery rate analysis for associations with genetically predicted intelligence

|  |  | **Univariable MR** | | **MVMR with education adjusted** | |
| --- | --- | --- | --- | --- | --- |
| **Class** | **Outcome** | ***p-value*** | **Result** | ***p-value*** | **Result** |
| Mental disorder | Obsessive-compulsive disorder | 4.1E-03 | TRUE | 1.01E-01 | FALSE |
| Mental disorder | Anorexia nervosa | 2.0E-05 | TRUE | 7.53E-01 | FALSE |
| Mental disorder | Bipolar disorder | 9.4E-01 | FALSE | - | - |
| Mental disorder | Schizophrenia | 1.5E-04 | TRUE | 1.46E-04 | TRUE |
| Mental disorder | Anxiety | 4.6E-02 | FALSE | - | - |
| Mental disorder | Posttraumatic stress disorder | 4.6E-01 | FALSE | - | - |
| Mental disorder | Major depressive disorder | 7.8E-01 | FALSE | - | - |
| Mental disorder | Insomnia | 6.4E-03 | TRUE | 8.60E-02 | FALSE |
| Mental disorder | Suicide attempts | 1.6E-03 | TRUE | 4.60E-02 | FALSE |
| Neurological disease | Alzheimer's disease | 1.9E-05 | TRUE | 1.67E-01 | FALSE |
| Neurological disease | Amyotrophic lateral sclerosis | 3.8E-01 | FALSE | - | - |
| Cardiovascular disease | Coronary artery disease | 2.6E-11 | TRUE | 9.37E-01 | FALSE |
| Cardiovascular disease | Atrial fibrillation | 3.8E-01 | FALSE | - | - |
| Cardiovascular disease | Heart failure | 2.0E-01 | FALSE | - | - |
| Cardiovascular disease | Total stroke | 3.7E-02 | FALSE | - | - |
| Cardiovascular disease | Any ischemic stroke | 2.6E-01 | FALSE | - | - |
| Cardiovascular disease | Large artery stroke | 4.1E-01 | FALSE | - | - |
| Cardiovascular disease | Small vessel stroke | 1.8E-02 | FALSE | - | - |
| Cardiovascular disease | Cardioembolic stroke | 3.7E-01 | FALSE | - | - |
| Cardiovascular disease | Intracerebral hemorrhage | 3.6E-02 | FALSE | - | - |
| Cancer | Breast cancer | 1.3E-01 | FALSE | - | - |
| Cancer | Breast cancer ER+ | 3.3E-01 | FALSE | - | - |
| Cancer | Breast cancer ER- | 1.6E-03 | TRUE | 9.32E-01 | FALSE |
| Cancer | Prostate cancer | 5.3E-01 | FALSE | - | - |
| Cancer | Lung cancer | 1.8E-02 | TRUE | 6.78E-01 | FALSE |
| Other disease | Type 2 diabetes | 1.3E-02 | TRUE | 2.39E-01 | FALSE |
| Other disease | Chronic kidney disease | 2.0E-01 | FALSE | - | - |
| Other disease | Fracture | 4.6E-01 | FALSE | - | - |
| Other disease | Gout | 1.0E+00 | FALSE | - | - |
| Other disease | Rheumatoid arthritis | 3.5E-03 | TRUE | 3.70E-02 | FALSE |
| Other disease | Inflammatory bowel disease | 2.4E-02 | FALSE | - | - |
| Other disease | Atopic dermatitis | 5.8E-01 | FALSE | - | - |
| Risk factor | Body mass index | 9.94E-07 | TRUE | 5.45E-01 | FALSE |
| Risk factor | Cigarettes per day | 1.62E-01 | FALSE | - | - |

**Supplementary Table 5.** Associations of genetically predicted education years with diseases based on the weighted median and MR-Egger

|  |  |  |  | **Weighted median method** | | | **MR-Egger regression** | | | |
| --- | --- | --- | --- | --- | --- | --- | --- | --- | --- | --- |
| **Class** | **Outcome** | **SNPs** | **Cochrane’ Q** | **OR** | **95% CI** | ***p*** | **OR** | **95% CI** | ***p*** | ***p_intercept_*** |
| Mental disorder | Obsessive-compulsive disorder | 663 | 805 | 1.67 | 1.12, 2.48 | 0.011 | 0.89 | 0.31, 2.56 | 0.825 | 0.081 |
| Mental disorder | Anorexia nervosa | 656 | 1090 | 1.83 | 1.53, 2.18 | 3.14E-11 | 1.48 | 0.89, 2.47 | 0.133 | 0.555 |
| Mental disorder | Bipolar disorder | 663 | 1443 | 1.40 | 1.17, 1.67 | 2.66E-04 | 1.31 | 0.75, 2.3 | 0.348 | 0.367 |
| Mental disorder | Schizophrenia | 663 | 1802 | 1.08 | 0.92, 1.27 | 0.348 | 0.98 | 0.55, 1.73 | 0.945 | 0.596 |
| Mental disorder | Anxiety | 654 | 621 | 1.78 | 1.28, 2.48 | 0.001 | 1.86 | 0.8, 4.31 | 0.148 | 0.917 |
| Mental disorder | Posttraumatic stress disorder | 663 | 714 | 0.89 | 0.56, 1.4 | 0.603 | 0.64 | 0.2, 2.02 | 0.449 | 0.445 |
| Mental disorder | Major depressive disorder | 659 | 1747 | 0.78 | 0.74, 0.83 | 3.63E-16 | 0.77 | 0.63, 0.94 | 0.010 | 0.878 |
| Mental disorder | Insomnia | 651 | 1212 | 0.74 | 0.69, 0.79 | 6.93E-19 | 0.83 | 0.68, 1.01 | 0.058 | 0.137 |
| Mental disorder | Suicide attempts | 591 | 646 | 0.63 | 0.49, 0.81 | 2.98E-04 | 1.70 | 0.91, 3.15 | 0.095 | 0.888 |
| Neurological disease | Alzheimer's disease | 663 | 669 | 0.72 | 0.61, 0.85 | 1.17E-04 | 0.71 | 0.47, 1.07 | 0.104 | 0.955 |
| Neurological disease | Amyotrophic lateral sclerosis | 663 | 672 | 0.89 | 0.72, 1.1 | 0.271 | 0.76 | 0.46, 1.26 | 0.283 | 0.443 |
| Cardiovascular disease | Coronary artery disease | 663 | 860 | 0.65 | 0.58, 0.72 | 1.40E-14 | 0.71 | 0.52, 0.97 | 0.034 | 0.422 |
| Cardiovascular disease | Atrial fibrillation | 663 | 1210 | 0.93 | 0.85, 1.01 | 0.101 | 1.00 | 0.76, 1.32 | 0.994 | 0.341 |
| Cardiovascular disease | Heart failure | 663 | 729 | 0.58 | 0.47, 0.71 | 1.75E-07 | 0.44 | 0.26, 0.73 | 0.001 | 0.512 |
| Cardiovascular disease | Total stroke | 663 | 828 | 0.73 | 0.66, 0.8 | 8.02E-11 | 0.81 | 0.62, 1.05 | 0.117 | 0.175 |
| Cardiovascular disease | Any ischemic stroke | 663 | 835 | 0.70 | 0.61, 0.79 | 9.51E-09 | 0.64 | 0.47, 0.89 | 0.007 | 0.858 |
| Cardiovascular disease | Large artery stroke | 663 | 765 | 0.63 | 0.47, 0.85 | 0.002 | 0.99 | 0.46, 2.15 | 0.989 | 0.082 |
| Cardiovascular disease | Small vessel stroke | 663 | 701 | 0.57 | 0.43, 0.75 | 9.22E-05 | 0.58 | 0.29, 1.14 | 0.114 | 0.916 |
| Cardiovascular disease | Cardioembolic stroke | 663 | 720 | 0.78 | 0.63, 0.98 | 0.029 | 0.88 | 0.49, 1.58 | 0.671 | 0.524 |
| Cardiovascular disease | Intracerebral hemorrhage | 510 | 524 | 0.42 | 0.22, 0.81 | 0.010 | 0.55 | 0.1, 2.93 | 0.487 | 0.648 |
| Cancer | Breast cancer | 663 | 1291 | 0.89 | 0.82, 0.97 | 0.005 | 0.84 | 0.66, 1.08 | 0.185 | 0.643 |
| Cancer | Breast cancer ER+ | 663 | 1187 | 0.95 | 0.86, 1.04 | 0.237 | 0.81 | 0.61, 1.09 | 0.164 | 0.381 |
| Cancer | Breast cancer ER- | 663 | 912 | 0.75 | 0.65, 0.87 | 1.07E-04 | 0.59 | 0.4, 0.86 | 0.007 | 0.250 |
| Cancer | Prostate cancer | 651 | 1350 | 1.11 | 1, 1.22 | 0.052 | 1.05 | 0.75, 1.47 | 0.786 | 0.758 |
| Cancer | Lung cancer | 657 | 679 | 0.53 | 0.43, 0.66 | 1.20E-08 | 0.53 | 0.31, 0.89 | 0.017 | 0.786 |
| Other disease | Type 2 diabetes | 663 | 2136 | 0.54 | 0.5, 0.59 | 1.69E-46 | 0.68 | 0.5, 0.94 | 0.019 | 0.088 |
| Other disease | Chronic kidney disease | 663 | 1016 | 0.86 | 0.77, 0.96 | 0.009 | 0.86 | 0.62, 1.19 | 0.364 | 0.765 |
| Other disease | Fracture | 663 | 908 | 0.99 | 0.92, 1.07 | 0.862 | 0.99 | 0.8, 1.23 | 0.935 | 0.720 |
| Other disease | Gout | 663 | 910 | 0.86 | 0.74, 1 | 0.052 | 0.74 | 0.48, 1.13 | 0.164 | 0.676 |
| Other disease | Rheumatoid arthritis | 659 | 1105 | 0.45 | 0.36, 0.57 | 2.33E-11 | 0.71 | 0.36, 1.39 | 0.318 | 0.281 |
| Other disease | Inflammatory bowel disease | 657 | 1359 | 0.70 | 0.6, 0.82 | 9.68E-06 | 0.99 | 0.59, 1.64 | 0.957 | 0.144 |
| Other disease | Atopic dermatitis | 663 | 692 | 1.05 | 0.85, 1.3 | 0.630 | 0.93 | 0.56, 1.56 | 0.797 | 0.594 |

CI indicates confidence interval; ER, estrogen receptor; OR, odds ratio; SD, standard deviation.

**Supplementary Table 6.** Associations of genetically predicted intelligence with diseases, body mass index and smoking

|  |  |  |  | **IVW-random effects method** | | | **Weighted median method** | | | **MR-Egger regression** | | | |
| --- | --- | --- | --- | --- | --- | --- | --- | --- | --- | --- | --- | --- | --- |
| **Class** | **Outcome** | **SNPs** | **Cochrane’ Q** | **OR** | **95% CI** | ***p*** | **OR** | **95% CI** | ***p*** | **OR** | **95% CI** | ***p*** | ***p_intercept_*** |
| Mental disorder | Obsessive-compulsive disorder | 178 | 203 | 1.55 | 1.15, 2.09 | 0.004 | 1.37 | 0.89, 2.11 | 0.158 | 3.54 | 0.88, 14.34 | 0.078 | 0.237 |
| Mental disorder | Anorexia nervosa | 174 | 391 | 1.45 | 1.22, 1.73 | 2.00E-05 | 1.42 | 1.18, 1.71 | 2.68E-04 | 3.28 | 1.43, 7.52 | 0.006 | 0.051 |
| Mental disorder | Bipolar disorder | 178 | 538 | 0.99 | 0.82, 1.21 | 0.944 | 0.99 | 0.81, 1.2 | 0.916 | 1.06 | 0.43, 2.61 | 0.900 | 0.885 |
| Mental disorder | Schizophrenia | 178 | 680 | 0.69 | 0.57, 0.83 | 1.49E-04 | 0.74 | 0.62, 0.89 | 0.001 | 1.01 | 0.41, 2.51 | 0.975 | 0.389 |
| Mental disorder | Anxiety | 175 | 182 | 1.28 | 1.00, 1.62 | 0.046 | 1.17 | 0.82, 1.66 | 0.394 | 0.66 | 0.19, 2.31 | 0.512 | 0.293 |
| Mental disorder | Posttraumatic stress disorder | 178 | 189 | 1.13 | 0.81, 1.57 | 0.464 | 1.16 | 0.71, 1.87 | 0.558 | 0.61 | 0.13, 2.85 | 0.533 | 0.424 |
| Mental disorder | Major depressive disorder | 178 | 601 | 0.99 | 0.93, 1.06 | 0.779 | 0.99 | 0.93, 1.05 | 0.788 | 0.93 | 0.69, 1.26 | 0.651 | 0.689 |
| Mental disorder | Insomnia | 178 | 366 | 0.92 | 0.87, 0.98 | 0.006 | 0.92 | 0.86, 0.99 | 0.018 | 0.92 | 0.7, 1.21 | 0.533 | 0.979 |
| Mental disorder | Suicide attempts | 164 | 154 | 0.77 | 0.65, 0.91 | 0.002 | 0.76 | 0.6, 0.97 | 0.026 | 0.88 | 0.41, 1.89 | 0.744 | 0.722 |
| Neurological disease | Alzheimer's disease | 178 | 293 | 0.72 | 0.61, 0.83 | 1.90E-05 | 0.74 | 0.62, 0.89 | 0.001 | 0.56 | 0.27, 1.16 | 0.121 | 0.508 |
| Neurological disease | Amyotrophic lateral sclerosis | 177 | 282 | 0.92 | 0.76, 1.11 | 0.378 | 0.91 | 0.74, 1.13 | 0.412 | 1.43 | 0.61, 3.35 | 0.408 | 0.297 |
| Cardiovascular disease | Coronary artery disease | 178 | 216 | 0.74 | 0.68, 0.81 | 2.60E-11 | 0.75 | 0.67, 0.85 | 2.42E-06 | 0.73 | 0.48, 1.11 | 0.145 | 0.940 |
| Cardiovascular disease | Atrial fibrillation | 178 | 314 | 0.97 | 0.89, 1.04 | 0.378 | 0.94 | 0.86, 1.04 | 0.227 | 0.79 | 0.55, 1.15 | 0.223 | 0.291 |
| Cardiovascular disease | Heart failure | 178 | 230 | 0.90 | 0.77, 1.06 | 0.199 | 0.92 | 0.74, 1.15 | 0.462 | 0.32 | 0.16, 0.66 | 0.002 | 0.005 |
| Cardiovascular disease | Total stroke | 177 | 230 | 0.92 | 0.85, 1.00 | 0.037 | 0.93 | 0.84, 1.02 | 0.133 | 0.97 | 0.67, 1.41 | 0.883 | 0.770 |
| Cardiovascular disease | Any ischemic stroke | 178 | 232 | 0.95 | 0.86, 1.04 | 0.258 | 0.95 | 0.83, 1.08 | 0.435 | 0.78 | 0.50, 1.21 | 0.270 | 0.377 |
| Cardiovascular disease | Large artery stroke | 178 | 202 | 0.91 | 0.73, 1.14 | 0.410 | 1.11 | 0.82, 1.51 | 0.498 | 1.03 | 0.37, 2.91 | 0.953 | 0.812 |
| Cardiovascular disease | Small vessel stroke | 178 | 206 | 0.78 | 0.63, 0.96 | 0.018 | 0.76 | 0.57, 1.01 | 0.061 | 0.68 | 0.26, 1.80 | 0.439 | 0.779 |
| Cardiovascular disease | Cardioembolic stroke | 178 | 231 | 0.92 | 0.77, 1.10 | 0.372 | 0.91 | 0.71, 1.15 | 0.421 | 1.00 | 0.42, 2.36 | 0.993 | 0.855 |
| Cardiovascular disease | Intracerebral hemorrhage | 137 | 190 | 0.54 | 0.31, 0.96 | 0.036 | 0.47 | 0.22, 1.00 | 0.049 | 2.17 | 0.13, 35.15 | 0.587 | 0.322 |
| Cancer | Breast cancer | 178 | 385 | 0.94 | 0.87, 1.02 | 0.129 | 0.90 | 0.83, 0.99 | 0.023 | 0.78 | 0.54, 1.12 | 0.174 | 0.290 |
| Cancer | Breast cancer ER+ | 178 | 346 | 0.96 | 0.88, 1.05 | 0.332 | 0.90 | 0.82, 1.00 | 0.041 | 0.82 | 0.54, 1.23 | 0.334 | 0.437 |
| Cancer | Breast cancer ER- | 178 | 216 | 0.84 | 0.76, 0.94 | 0.002 | 0.87 | 0.76, 1.00 | 0.057 | 0.55 | 0.34, 0.89 | 0.017 | 0.077 |
| Cancer | Prostate cancer | 174 | 362 | 0.97 | 0.88, 1.07 | 0.529 | 1.02 | 0.91, 1.14 | 0.732 | 1.00 | 0.64, 1.58 | 0.989 | 0.878 |
| Cancer | Lung cancer | 175 | 222 | 0.82 | 0.69, 0.97 | 0.018 | 0.84 | 0.68, 1.04 | 0.117 | 1.23 | 0.55, 2.76 | 0.617 | 0.311 |
| Other disease | Type 2 diabetes | 178 | 849 | 0.87 | 0.77, 0.97 | 0.013 | 0.85 | 0.78, 0.93 | 0.001 | 1.14 | 0.67, 1.92 | 0.635 | 0.299 |
| Other disease | Chronic kidney disease | 178 | 270 | 0.94 | 0.85, 1.03 | 0.201 | 0.92 | 0.82, 1.04 | 0.172 | 0.81 | 0.52, 1.25 | 0.340 | 0.486 |
| Other disease | Fracture | 178 | 307 | 0.97 | 0.91, 1.05 | 0.462 | 0.96 | 0.88, 1.05 | 0.352 | 0.89 | 0.65, 1.23 | 0.491 | 0.589 |
| Other disease | Gout | 178 | 232 | 1.00 | 0.89, 1.13 | 0.998 | 1.01 | 0.86, 1.2 | 0.871 | 0.93 | 0.53, 1.63 | 0.812 | 0.806 |
| Other disease | Rheumatoid arthritis | 176 | 264 | 0.76 | 0.63, 0.91 | 0.003 | 1.00 | 0.79, 1.27 | 1.000 | 0.38 | 0.16, 0.93 | 0.036 | 0.126 |
| Other disease | Inflammatory bowel disease | 173 | 427 | 0.81 | 0.69, 0.95 | 0.011 | 0.78 | 0.66, 0.93 | 0.006 | 0.51 | 0.24, 1.08 | 0.079 | 0.215 |
| Other disease | Atopic dermatitis | 178 | 220 | 1.05 | 0.89, 1.23 | 0.576 | 0.95 | 0.76, 1.18 | 0.621 | 1.13 | 0.52, 2.45 | 0.754 | 0.840 |
| Risk factor | Body mass index | 178 | 2393 | 0.88 | 0.83, 0.92 | 9.94E-07 | 0.94 | 0.91, 0.98 | 0.001 | 0.90 | 0.71, 1.15 | 0.403 | 0.819 |
| Risk factor | Cigarettes per day | 178 | 404 | 0.95 | 0.89, 1.02 | 0.162 | 0.97 | 0.90, 1.04 | 0.367 | 0.89 | 0.65, 1.22 | 0.471 | 0.671 |

CI indicates confidence interval; ER, estrogen receptor; IVW, inverse variance weighted; OR, odds ratio; SD, standard deviation.

**Supplementary Table 7.** Associations of genetically predicted intelligence with outcomes after adjustment for genetically predicted education level

| **Class** | **Outcome** | **OR** | **95% CI** | ***p*** |
| --- | --- | --- | --- | --- |
| Mental disorder | Obsessive-compulsive disorder | 1.68 | 0.9, 3.13 | 0.101 |
| Mental disorder | Anorexia nervosa | 1.06 | 0.74, 1.51 | 0.753 |
| Mental disorder | Schizophrenia | 0.46 | 0.31, 0.69 | 1.46E-04 |
| Mental disorder | Insomnia | 1.11 | 0.98, 1.26 | 0.086 |
| Mental disorder | Suicide attempts | 0.70 | 0.49, 0.99 | 0.046 |
| Neurological disease | Alzheimer's disease | 0.80 | 0.58, 1.1 | 0.167 |
| Cardiovascular disease | Coronary artery disease | 0.99 | 0.83, 1.18 | 0.937 |
| Cancer | Breast cancer ER- | 1.01 | 0.81, 1.25 | 0.932 |
| Cancer | Lung cancer | 0.93 | 0.66, 1.31 | 0.678 |
| Other disease | Type 2 diabetes | 1.15 | 0.91, 1.45 | 0.239 |
| Other disease | Rheumatoid arthritis | 1.49 | 1.02, 2.16 | 0.037 |
| Risk factor | Body mass index | 0.97 | 0.87, 1.08 | 0.545 |

**Supplementary Table 8.** Associations of education level with body mass index and smoking in the weighted median and MR-Egger models

| **Risk factor** |  |  | **Weighted median method** | | | **MR-Egger regression** | | | |
| --- | --- | --- | --- | --- | --- | --- | --- | --- | --- |
|  | **SNPs** | **Cochrane’ Q** | **Change** | **95% CI** | ***p*** | **Change** | **95% CI** | ***p*** | ***p_intercept_*** |
| Body mass index | 663 | 5619 | -0.28 | -0.31, -0.25 | 2.80E-85 | -0.27 | -0.41, -0.12 | 4.35E-04 | 0.319 |
| Cigarettes per day | 663 | 1354 | -0.32 | -0.39, -0.25 | 9.86E-19 | -0.25 | -0.47, -0.03 | 2.84E-02 | 0.165 |

CI, confidence interval.

**Supplementary Table 9.** Comparison of the results of the present MR study with those of meta-analysis of observational studies

| **Outcome** | **Meta-analysis** | | | **MR analysis** | | |
| --- | --- | --- | --- | --- | --- | --- |
|  | **N** | **OR** | **95% CI** | **N** | **OR** | **95% CI** |
| Major depressive disorder | 50988 | 0.67 | 0.58, 0.78 | 500199 | 0.72 | 0.68, 0.76 |
| Alzheimer's disease | >5000 | 0.77 | 0.71, 0.83 | 63926 | 0.70 | 0.63, 0.78 |
| Suicide attempts | 2954 | 1.24 | 0.80, 1.93 | 50264 | 0.56 | 0.48, 0.67 |
| Amyotrophic lateral sclerosis | NA | 0.49 | 0.44, 0.55 | 36052 | 0.92 | 0.80, 1.05 |
| Posttraumatic stress disorder | 68685 | 0.81 | 0.76, 0.86 | 200000 | 0.99 | 0.72, 1.35 |
| Coronary artery disease | NA | 0.74 | 0.64, 0.85 | 184305 | 0.56 | 0.46, 0.67 |
| Heart failure | 6308006 | 0.60 | 0.53, 0.68 | 395034 | 0.51 | 0.45, 0.59 |
| Total stroke | 2737522 | 0.74 | 0.70, 0.79 | 521612 | 0.68 | 0.63, 0.73 |
| Breast cancer | >10 million | 1.22 | 1.13, 1.32 | 228951 | 0.89 | 0.83, 0.96 |
| Prostate cancer | >14736 | 0.63 | 0.53, 0.75 | 140306 | 1.10 | 1.01, 1.21 |
| Lung cancer | 2562221 | 0.61 | 0.56, 0.67 | 27209 | 0.49 | 0.43, 0.56 |
| Type 2 diabetes | 255445 | 0.71 | 0.67, 0.75 | 898130 | 0.52 | 0.48, 0.57 |
| Chronic kidney disease | 193226 | 0.83 | 0.77, 0.89 | 480698 | 0.90 | 0.82, 0.98 |
| Body mass index | 164049 | 0.89 | 0.86, 0.94 | 694649 | 0.71 | 0.69, 0.74 |

CI indicates confidence interval; MR, Mendelian randomization. The effect size of MR was based on univariable inverse-variance weighted method. The effect size of meta-analysis was derived by comparing higher education level with lower education, except for body mass index in a linear association with education level.

**Supplementary Figure 1.** Study design and hypothesis

**
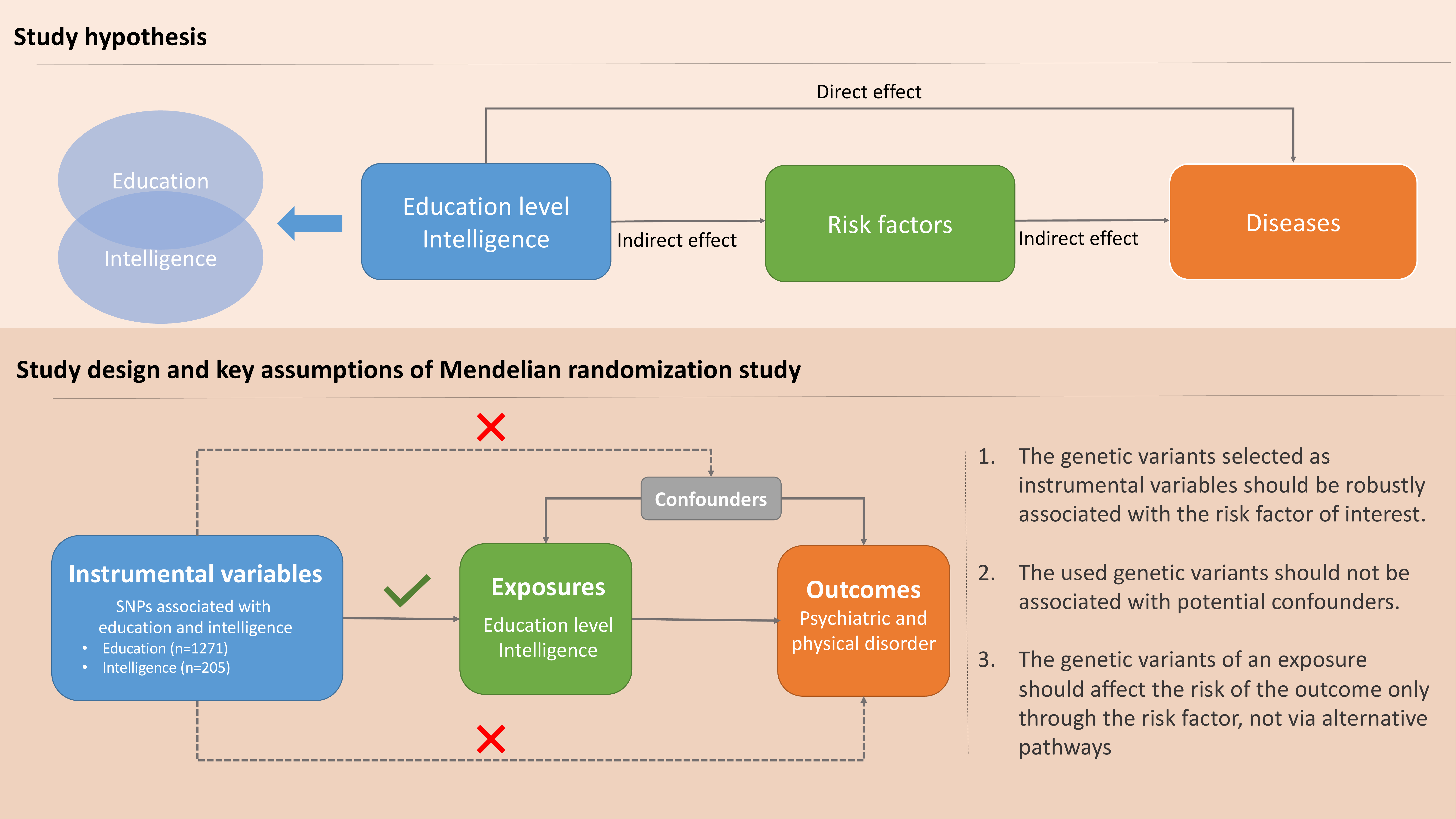
**

We assumed that education level (years of education) and intelligence influenced diseases mostly via risk factors (indirect effect) and partly not via risk factors (direct effect). There was an overlapping between education level and intelligence given that we found a bidirectional causal association between education level and intelligence. Thus, we used multivariable MR analysis method to distinguish the effects of education from intelligence.
